# The current state and future direction of DoD gut microbiome research: a summary of the first DoD gut microbiome informational meeting

**DOI:** 10.1186/s40793-018-0308-0

**Published:** 2018-03-20

**Authors:** Steven Arcidiacono, Jason W. Soares, J. Philip Karl, Linda Chrisey, C. P. T. Blair C. R. Dancy, Michael Goodson, Fredrick Gregory, Rasha Hammamieh, Nancy Kelley Loughnane, Robert Kokoska, C. A. P. T. Mark Riddle, Keith Whitaker, Kenneth Racicot

**Affiliations:** 1 Natick Soldier Research Development & Engineering Center, Boston, MA USA; 2 US Army Research Institute of Environmental Medicine, Boston, MA USA; 3 Office of Naval Research, Arlington, VA USA; 4 US Army Center for Environmental Health Research, Annapolis, MD USA; 5 Air Force Research Laboratory, Columbus, OH USA; 6 US Army Research Office, Annapolis, MD USA; 7 Naval Medical Research Center, Annapolis, MD USA; 8 US Army Research Laboratory, Annapolis, MD USA

**Keywords:** Microbiome, Performance, Warfighter, Nutrition, Stressors, Biomarkers, Health, Disease, PTSD, Gut-brain axis

## Abstract

The gut microbiome is increasingly recognized as integral to human health, and is emerging as a mediator of human physical and cognitive performance. This has led to the recognition that US Department of Defense (DoD) research supporting a healthy and resilient gut microbiome will be critical to optimizing the health and performance of future Warfighters. To facilitate knowledge dissemination and collaboration, identify resource capabilities and gaps, and maximize the positive impact of gut microbiome research on the Warfighter, DoD partners in microbiome research participated in a 2-day informational meeting co-hosted by the Natick Soldier Research, Engineering and Development Center (NSRDEC) and the US Army Research Institute of Environmental Medicine (USARIEM) on 16–17 November 2015. Attendee presentations and discussions demonstrated that multiple DoD organizations are actively advancing gut microbiome research. Common areas of research included the influence of military-relevant stressors on interactions between the microbiome and Warfighter biology, manipulation of the microbiome to influence Warfighter health, and use of the microbiome as a biomarker of Warfighter health status. Although resources and capabilities are available, they vary across laboratories and it was determined that centralizing certain DoD capabilities could accelerate progress. More significantly, the meeting created a foundation for a coordinated gut microbiome and nutrition research program aligning key DoD partners in the area of microbiome research. This report details the presentations and discussions presented during the 1st DoD Gut Microbiome Informational Meeting.

## Introduction

The human body hosts trillions of microorganisms, whose collective genetic composition is collectively known as the human microbiome [1–3]. The human gut microbiome comprises a diverse, dense and active microbial ecosystem, also known as a microbiota, that resides in the gastrointestinal tract [1] and largely co-exists in a mutualistic relationship with the host. The composition and activity of gut microbes are modulated by changes in host diet and physiology while, in turn, gut microbes support immune health, deter pathogen invasion, regulate central and enteric nervous system activity, and generate beneficial nutrients and metabolites [4–6]. These beneficial relationships, however, can be perturbed by environmental stressors that directly or indirectly modulate the gut microbiome, thereby resulting in a state of dysbiosis that has been implicated in the development of acute health decrements, such as systemic inflammation [7], increased susceptibility to illness and infection [8], cognitive decrements [9, 10] and the development and/or persistence of multiple chronic diseases [8].

The launch of the Human Microbiome Project in 2007, supported with a $173 million investment from the NIH Common Fund (2007–2012) to advance human microbiome research, demonstrates government recognition of the microbiome’s potential importance to human health. The significance of microbiome-related research to global health priorities was further accentuated in May 2016, with the announcement of the White House National Microbiome Initiative [11] to accelerate and coordinate efforts to explore the role of the microbiome in the environment and in human health and disease. Similarly, there is growing recognition within the US Department of Defense (DoD) that research supporting a healthy and resilient gut microbiome will be critical for optimizing the health and performance of future Warfighters [12]. Warfighters are commonly exposed to degrading factors that compromise physical and cognitive performance such as sleep loss, suboptimal nutrition, environmental extremes, and prolonged physical exertion. Understanding the extent to which these factors degrade Warfighter performance, determining the underlying mechanisms, and identifying mitigation and resiliency strategies is a central theme within multiple recent DoD research and development directives [13–17]. The gut microbiome is emerging as one factor that may mediate the effects of these stressors on Warfighter health and performance. Since the gut microbiome is integral to health, but also malleable and strongly influenced by the host diet, nutrition-based interventions could provide novel, low-cost strategies for optimizing Warfighter health and performance through modulation of the gut microbiome.

The Natick Microbiome Interest Group (NMIG) is a recently formed research cluster exploring interactions between nutrition and gut microbiome to understand influence on Warfighter health and performance. The group is comprised of members from the US Army Research Institute of Environmental Medicine (USARIEM), the Combat Feeding Directorate (CFD) and Warfighter Directorate (WD), US Army Natick Soldier Research, Development and Engineering Center (NSRDEC), all located at Natick Soldier Systems Center (NSSC). Upon its formation in 2014, the NMIG recognized that although multiple DoD organizations were actively supporting gut microbiome-related research efforts, a coordinated gut microbiome and nutrition research program was absent. The group envisioned that enhancing coordination and information exchange across organizations conducting microbiome research could lead to more efficient use of resources and avoid duplication of efforts as DoD nutrition and gut microbiome research developed. To realize this goal, the NMIG hosted an informational meeting at NSSC on 16–17 November 2015 with the objective of aligning key DoD partners in multiple areas of microbiome research. The meeting goals included facilitating collaboration amongst scientists, identifying gaps within DoD gut microbiome research, enhancing knowledge sharing and coordination of research efforts, and identifying currently available resources and capabilities. Attendees represented multiple government organizations conducting microbiome research. On Day One of the event, invited DoD scientists presented an overview of gut microbiome research and areas of interest within their organizations (Table 1). During Day Two, four breakout sessions allowed for deep dives into subject areas relevant to identifying DoD nutrition and gut microbiome research gaps and needs. This report summarizes the discussion and conclusions of the 1st DoD Gut Microbiome Informational Meeting.

**Table 1:** DoD organizations and their general interests related to gut microbiome research

Organization	Interests
Army Research Office	Funding fundamental research toward understanding inherent interactions and downstream effects of various microbial communities including the gut microbiome
Army Research Laboratory	Understanding how gut microbiome influences human behavior, capability enhancement and human-system interaction, waste-to-energy conversion through metabolic pathway engineering of gut-derived microbiota
US Army Research Institute of Environmental Medicine	Conducts research providing a biomedical science basis for developing new rations, menus, policies and programs that enable Warfighter health-readiness and optimal performance; microbiome interests include interactions between military-relevant stressors, diet, and the gut microbiome, particularly within austere environments
US Army Center for Environmental Health Research	Molecular events associated with disease progression; microbiome as a tool for monitoring Warfighter exposure to environmental toxicants
Naval Medical Research Center	Deployment health implications of gastrointestinal infection, the microbiome and both acute and chronic disease
Office of Naval Research	Funding programs to understand the effects of certain behavioral and environmental stressors on a host and its gut microbiota, with an emphasis on deducing the role the gut microbiota may play in mediating psychological, cognitive and physiological effects of such exposures
US Army Natick Soldier Research, Development and Engineering Center	In vitro studies to determine military-relevant stressor effects on the microbiome and host to inform clinical trial design; elucidation of mechanistic knowledge of dietary input biotransformation by gut bacteria; optimizing combat feeding rations; performance nutrition
US Air Force Research Laboratory	Influence human performance in a non-invasive manner (cognition, anxiety, stress)
Walter Reed Army Institute of Research	Biomedical research that delivers lifesaving products including knowledge, technology and medical material that sustain the combat effectiveness of the Warfighter
Uniformed Services University of the Health Sciences	Nutrition, gastroenterology; Consortium for Health and Military Performance: research to improve service member performance in the field and returning to duty; animal studies
Naval Surface Warfare Center Dahlgren Division	Understanding influence of native and engineered biological threats on human health

### Day 1: Invited presentations

Dr. Robert Kokoska (Army Research Office (ARO) Microbiology Program Manager) presented an overview of extramural basic research programs sponsored by the Life Sciences Division of the US Army Research Laboratory (ARL). Dr. Kokoska described ARO programs as contributing to our understanding of the characterization, inherent interactions and downstream effects of various microbial communities, including the gut microbiome. The ARO Program in Microbiology, managed by Dr. Kokoska, has a primary strategic focus on the analysis and engineering of microbial communities. Single Investigator grants and Multidisciplinary University Research Initiative (MURI) awards managed under this program address the challenges of establishing functional links between species with divergent roles within a microbial consortium, understanding the role of spatial order within an interactive microbial consortia and the development of tools, methodologies and engineered systems that can effectively control and analyze the physical and biochemical characteristics of both simple and complex microbial communities. Within this program, research efforts that provide “bottom-up” approaches, i.e., those that examine simple well-ordered communities, hold the promise of uncovering fundamental interactive and control principles within a consortium that can aid our understanding of the forces that drive community structure and function.

In contrast to more controllable “bottom-up” approaches, most studies of the gut microbiome take on more data-rich “top-down” approaches toward untangling the myriad interactions within this highly complex natural community in order to infer functional output from the microbial consortia. The downstream functional effects of the gut microbiome are themselves highly complex and networked. For example, it has been theorized that there exists a bi-directional link between the gut microbiome and the brain, in which neural, hormonal, immunological and microbial signaling pathways are networked. This link between gut microbes and brain function has thus drawn technical and programmatic interest from the Neurophysiology of Cognition Program at ARO Life Sciences managed by Dr. Frederick Gregory. Dr. Gregory’s program is focused on the molecular, cellular and systems-level neural codes underlying human perception and behavior. In line with both the ARO Microbiology and Neurophysiology of Cognition program interests, it is thought that nutritional inputs and physical stressors can affect the composition of the gut microbiome in a manner that influences these various dynamic interactions between the gut and the brain, ultimately affecting cognition and behavior. The multi-layered complexity of interactions between the gut microbiome and the networked physiology, including neural and immune dynamics, confounds our ability to establish testable hypotheses that can point the way toward causative rather than correlative effects within this interactive network. Thus, a need has been identified to establish a systems-level mathematical modeling framework that can better guide our understanding of the performance of the microbiome-gut-brain axis. To meet this challenge, Dr. Gregory and Dr. Kokoska have teamed to establish an Army MURI topic that looks towards the development of a layered, cellular and system-level model that postulates cognitive and behavioral control of integrated neural, endocrine and immune interactions by commensal gut microorganisms in response to nutrition and physical stress. It is envisioned that the ability to inform and refine the model with metabolic data will generate multiscale predictions, constrain empirical approaches, and direct hypothesis-driven research toward an understanding of the causative linkages within the gut-brain axis.

Dr. Rasha Hammamieh presented an overview of Integrative Systems Biology (ISB) Program at US Army Center for Environmental Health Research (USACEHR), where they are trying to characterize molecular events associated with disease progression and identify pathways and networks to diagnose and predict the course of impending illness. This is carried out using clinical and patho-psychophysiological information with multi-omics and phenotypic readouts. ISB has done extensive work with a multi-core Post Traumatic Stress Disorder (PTSD) group and have used a cohort of PTSD+/− volunteers to identify epigenomic and genomic changes unique to PTSD individuals. An essential project is a study to understand the regulation of the microbiome profile in response to traumatic stress. In a mouse model simulating features of PTSD [18], the ISB group showed that simultaneous perturbed functioning of multiple organ systems (e.g., brain, heart, intestine, liver) that can produce injuries that lead to chronic metabolic changes associated with PTSD [19]. The metabolomics analysis showed altered gut-derived metabolites in plasma at 24 h post-stressor exposure and these remained altered up to 4 weeks after stressor withdrawal.

A recent review of the literature has demonstrated that gut bacteria strongly influence the metabolic, immune, endocrine, peripheral and central nervous systems. These gut-brain interactions are considered as bidirectional with major consequences for outcome of the disease. Considering the role of microbiome in psychiatric disorders, the ISB team also utilized fecal samples from the longitudinal study using the established rodent model simulating aspects of PTSD. The ISB group’s profiling included sequencing variable regions 3 and 4 of the 16S rRNA gene from fecal samples collected every day where subject mice were exposed to aggressor mice for 10 days. Bacterial community analysis included looking at community composition using Quantitative Insights into Microbial Ecology pipelines [20]. The analyses indicated that aggressor exposure did not impact the alpha diversity among samples at different time points. However, differences in *Firmicutes* *and* *Verrucomicrobia* populations in aggressor-exposed mice were observed as early as 24 h. Further, *Oscillospira*, *Lactobacillus*, *Akkermansia* and *Ruminococcus* were identified as the top four genera that were changed between control and aggressor-exposed mice. They are looking further into the functional pathway predictions based on bacterial composition at a particular time point using Phylogenetic Investigation of Communities by Reconstruction of Unobserved States [21], with preliminary indications that some changes in key metabolic regulators can be a consequence of the microbiome dysregulation in the presence of stress. These studies will provide new insights into how the microbiome changes upon stress exposure, although deeper analysis into the structure of the microbiome is required to identify the species level classification. Further, it is important to study mucosal versus luminal microbiome to determine the role in functional consequence of the disease given that differences between responses of the mucosal and luminal microbiomes to stress have been reported [22].

Drs. Keith Whitaker and Justin Brooks presented an overview of the ARL Human Science Campaign, with a specific focus on how the gut microbiome influences human behavior, capability enhancement, and human-system interaction. Dr. Whitaker and Dr. Brooks asserted that in order to achieve the goal of maximizing the effectiveness of Warfighters physically, perceptually, and cognitively, the Army must invest in understanding and engineering the human-associated microbiome as a component of inter- and intra-individual variability in performance. ARL’s intramural investment in the Human Sciences includes a diverse set of priorities centered on the integration of a range of technologies and concepts into multi-scale, human-centric research and advanced technology development. Within that context, there is an open question about the relative contribution of the gut microbiome, as compared to other sources of performance variability, to Warfighter performance. Throughout ARL’s intramural research portfolio, there are research and technology development projects that can be leveraged to support the Army’s need for altering the human gut microbiome, even if that is not the primary objective. For example, Dr. Whitaker leads a high-risk research project on the bioengineering of extracellular vesicles with the intention of specifically, temporarily and reversibly altering cellular activity to inhibit inflammation after a traumatic brain injury. Extracellular vesicles are produced by many different cells types, including the bacteria of the human gut, and these carry nucleic acids and proteins into receptive cells. The ability to manipulate the contents and characteristics of these nano-sized vesicles may provide new biotechnology to influence the interaction of the human-microbiome system. On a larger scale, one of ARL’s newest programs, Continuous Multi-faceted Soldier Characterization for Adaptive Technologies (CMSCFAT), is focused on characterizing how the relationship between behavioral, physiological, environmental, and task-based factors drive and may predict variability in task performance in real-world environments. Recent advancements in our understanding of how microbial metabolism (particularly in the gut) influences host factors strongly suggest that the microbiota may have a profound effect on human performance, state, and emotion. As part of CMSCFAT, ARL will encourage and support internal research proposals that examine the gut microbiome as a potential source of behavioral and performance variability within the context of other physiological and environmental variables. Currently, research projects that have collected whole blood samples may be used as part of this effort to investigate microbial metabolites that have entered through the enteric system, which will then be related to behavioral and physiological variability. While research in this area is nascent within ARL, there is growing interest in how the gut microbiome may affect human performance and mechanisms that are in place to support the development of this line of research.

**Fig. 1:**
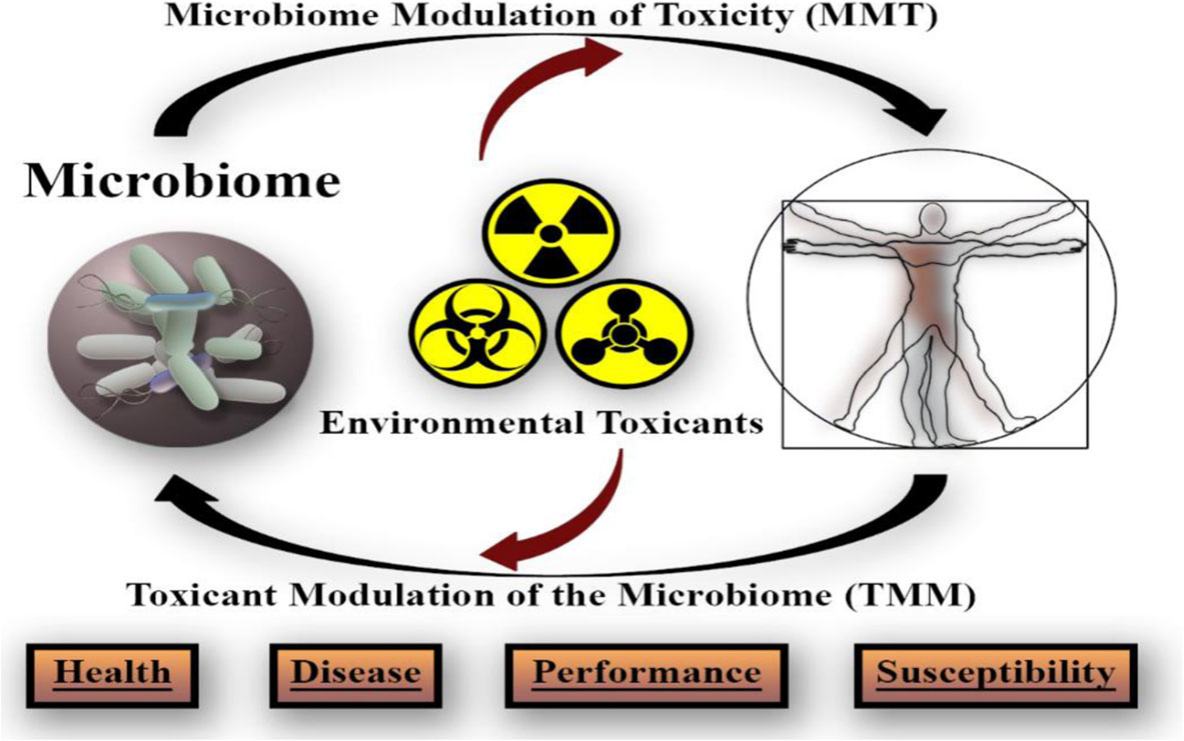
The Microbiome-Toxicant Dynamic

Captain Blair Dancy, PhD of USACEHR, located at Fort Detrick, Maryland, described how efforts within USACEHR’s Environmental Health Program (EHP) are currently advancing a multi-institutional initiative to explore the utility of the microbiome as a tool for monitoring Warfighter exposure to environmental toxicants. CPT Dancy then outlined the rationale underlying microbiome research efforts underway or planned within the EHP. In the context of an environmental exposure, the microbiome represents the very first interface between an externally encountered chemical and the toxicant-manifested clinical disease [23]. This initial interaction between a toxicant and the microbiome provides for a valuable opportunity to develop microbiome-based biomarkers of exposure and susceptibility that could be used as early indicators of exposure or employed to provide longitudinal exposure surveillance. Furthermore, the relationship between environmental toxicants and the microbiome is dynamic. Not only is the microbiome subject to the effects of toxicants (Toxicant Modulation of the Microbiome (TMM)), but toxicants are subject to modification by the microbiome, potentially resulting in altered toxicity profiles for compounds (Microbiome Modulation of Toxicity (MMT)) (Fig. 1).

Initial efforts at the USACEHR to explore the role that environmental toxicants have on the microbiome have used fecal pellets collected from conventional rats, pre- and post-exposure to toxicants, including sodium arsenite, cadmium chloride, sodium dichromate, nickel chloride, and cobalt chloride. Fecal samples were analyzed through academic and industrial collaborators using 16S rRNA analysis, small-molecule metabolomics and lipidomics. These analyses are expected to reveal panels of specific bacterial taxonomic groups and small molecules whose perturbations are indicative of toxicant exposure. To better address the utility of the human microbiome as an exposure surveillance tool, the USACEHR is currently using a humanized-gut-microbiota-mouse as a toxicant exposure model. These mice, which are born germ free, are provided a human-gut flora through a procedure known as fecal microbiota transplantation. The USACEHR has partnered to obtain a screened source of human fecal material and deliver it into a commercial source of germ-free animals, and has currently conducted several exposures utilizing the humanized-gut-microbiota mice. Fecal material from the humanized-gut-microbiota mouse exposures will be longitudinally collected over the course of the experiment’s duration. These fecal samples will then be analyzed using next-generation sequencing, in collaboration with the U.S. Army Medical Research Institute of Infectious Diseases (USAMRIID), and metabolomics tools will be utilized to identify biomarkers of exposure. The USACEHR is also currently partnering with other organizations, including the U.S. Air Force Research Laboratory (AFRL), 711th Human Performance Wing, to explore the effect that sodium dichromate and engineered nanomaterials have upon the microbiome. Additionally USACEHR has partnered with the U.S. Army Public Health Center for completing a review on the role of the microbiome in chemical toxicity, and to host a multi-organizational meeting at the Defense Health Headquarters to evaluate the microbiome role in health risk assessment. Finally, USACEHR also partnered with the Johns Hopkins University Applied Physics Laboratory, a University Applied Research Center (UARC), and the Massachusetts Institute of Technology Lincoln Laboratory, a Federally Funded Research and Development Center (FFRDC), to develop tools to explore the host response to toxicant exposures and to provide new sequencing and bioinformatics tools to profile bacterial communities using 16S rRNA sequencing, shotgun metagenomic datasets, and mobile genetic elements.

**Fig. 2:**
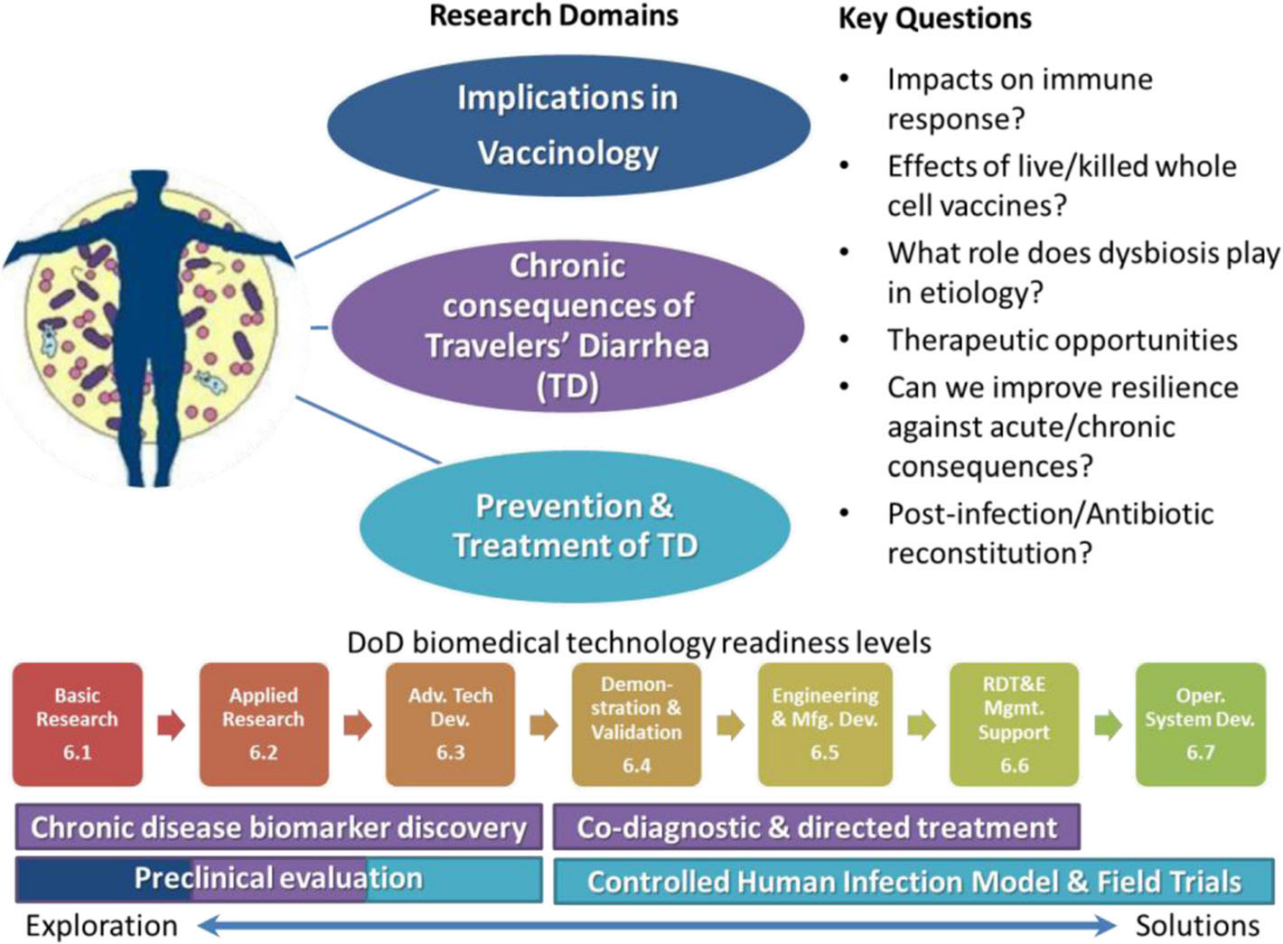
Schematic representation of NMRC microbiome research and key questions currently under consideration. Horizontal bars show scientific tasks associated with the research domains vaccinology (blue), chronic consequences (purple), and TD treatment and prevention (turquoise) and what technology readiness levels they fit. 6.1, 6.2 etc. are DoD designations for the various technology levels

CAPT Mark Riddle, MD of the Naval Medical Research Center (NMRC) presented an overview of research efforts, gaps and potential materiel solutions relative to deployment health implications of gastrointestinal infection, on the microbiome and acute/chronic disease. CAPT Riddle directs the US Military program on research related to vaccine development of bacterial enteric infections, as well as discovery and translational efforts in the area of intestinal disease biomarkers and optimized treatment strategies. The areas explored in his overview included implications on prevention and treatment of acute travelers’ diarrhea, the chronic consequences of these common deployment health infections, and implications in vaccinology (schematic overview shown in Fig. 2).

Acute enteric infection during deployment is ranked as the 2nd leading infectious disease threat to military service members and is a significant contribution of morbidity in the United States [24]. On average, 29% per month become afflicted with a diarrheal illness during deployment in the developing world, and the majority of these infections are due to bacterial etiologies. While vaccines against the leading causes of bacterial diarrheas (*Campylobacter jejuni*, *Shigella* spp. and enterotoxigenic *E. coli*) are under development based on DoD requirements and the Military Infectious Diseases Research Program, these are long-term solutions while short- and intermediate-term solutions are needed to mitigate this disease burden and its effect on mission readiness. As our understanding of the importance of the human microbiome in health and disease has advanced, interest in the use of nonpathogenic bacteria and yeast (probiotics), as well as nutrients which enhance the growth of favorable microbes (prebiotics) is an appealing concept, given the potential ease of use, and, being natural product derivatives, appear to be a safe alternative to antibiotics [25]. Mechanisms including competition for nutrients, competition for adhesion sites, direct antagonism, and immune stimulation have been explored and offer a strong rationale to support enhancement of the host-microbiome as a “gut shield system” in the Warfighter. However, despite the attractiveness of this strategy, to date clinical studies that have evaluated only a few probiotic/prebiotic products provide results that are too variable to support current use [26, 27]. Findings from relevant studies have also been difficult to interpret due to differences in the species, formulations and dosages of probiotics studies, and also due to methodological problems within the studies themselves (i.e., poor compliance, recall bias). Complicating this matter is the fact that these products, which have been evaluated for the indication of acute enteric illness prevention, have not undergone the necessary FDA regulated trials required for a product to be used for a preventative indication under Force Health Protection regulations. However, despite limitations in the current evidence base, a commercially available prebiotic has demonstrated some evidence of efficacy [28], and researchers at the Naval Medical Research Center are actively pursuing an Investigational New Drug clinical trial under industry sponsorship. In addition to the acute symptoms and morbidity associated with diarrhea during deployment, a growing body of literature from both military and non-military populations is defining a significant burden of chronic gastrointestinal disorders following acute enteric infections [29, 30]. Further, the recognition of multi-drug resistant organism (MDRO) acquisition among travelers with diarrhea independent of antibiotics being used, calls for further efforts to consider how to enhance either colonization resistance against such MDRO or displacement of these colonizers during or post travel or deployment [31]. While gaps remain in our complete understanding of these complex disorders, gut barrier integrity, inflammation, and dysbiosis are hallmarks of disease, which can result in life-long disability. Researchers from the NMRC have begun discovery efforts in collaboration with professional society and industry support to try and understand basic pathobiology underpinning individual susceptibility and diagnostic markers that may eventually lead to precision medicine directed strategies for disease prevention, interception or treatment [32, 33]. Interesting observations were discussed related to the host-microbiome and vaccinology. For example, recent studies have shown how the host microbiome is an important component in the host response to seasonal influenza vaccination [34] and how interference of induction of functional antibody responses to an experimental human HIV-1 vaccine was associated with cross-reacting microbiota antibodies [35]. These early observations highlight the importance of understanding how the microbiome may impact the variation in host responses, specifically to enteric vaccines under development and the impact of enteric vaccines on the host-microbiome. Finally, CAPT Riddle led a discussion on the challenges related to lack of specified research priorities and funding programs to address these gaps, but noted the incredible opportunity to uniquely study these important questions though active field site studies, biorepositories, and relevant small animal models.

Dr. Linda Chrisey (Program Officer, Office of Naval Research (ONR) Gut Microbiology for Warfighter Resilience Program) provided a background and objectives of ONR programs. The fairly young basic research program, is primarily funded through ONR’s Basic Research Challenge program (FY14 start) and an FY15 Multidisciplinary University Research Initiative topic (late FY15 start). Dr. Chrisey provided background for her programs by explaining that the commensal bacteria, fungi and viruses that comprise the gut microbiome, have tremendous influence on the development of the brain, central and enteric nervous systems; mucosal immune system and protection against pathogens, perception of pain; extraction of nutrients from food and distribution of fat; as well as the microbiome’s direct role in several diseases. Thus, it is critically important to enhance our understanding of this ‘microbial organ’ with a focus on its role in response to external stressors that can affect human resilience. She elaborated by noting that specific roles for the gut microbiome in emotional behavior, anxiety, memory and cognitive function, and neuromodulator expression have been documented, as well as supporting a role for the gut microbiome in bi-directional signaling via the gut-brain-axis, the hippocampal-pituitary axis, and for overall homeostasis. Of particular interest to ONR is the study of behavioral stressors (e.g., fear, anxiety, social crowding) and environmental stressors (e.g., environmental shifts in altitude, temperature, time zone, round-the-clock work shifts, altered circadian rhythms, fatigue, sporadic nutrition, pain, anticipatory anxiety, fear), to begin to functionally link specific microbes to observed host responses. Dr. Chrisey defined the objective of the ONR program as aiming to understand the effects of certain behavioral and environmental stressors on a host and its gut microbiota, with an emphasis on deducing the role the gut microbiota may play in mediating psychological, cognitive and physiological effects of such exposures. More specifically “Does the gut microbiome play a role in host response to physical and psychosocial stressors, and if so, can we envision ways to manipulate the microbiome to minimize negative responses?” The potential impact of the program is to develop the capability to increase Warfighter resilience to stressors via manipulation of the gut microbiota, such as using pre−/pro- or antibiotic prophylaxis or treatments. Such manipulation of the host-gut microbiota may allow us to enhance Warfighter fitness and resilience to these stressors. The program will attempt to study analogs of these stressors for which solid animal models and useful paradigms for human research exist and determine the role (if any) of the gut microbiota in the host response. The program will: a) characterize the psychological, physiological and cognitive responses of the host and gut microbiome following exposure to various stressors; b) ideally capture system response from metabolites/small signal molecules to whole organism(s); c) ascertain if specific gut microbial community members, metabolites or functions play a role in the observed stress response; d) utilize established animal models, assays, and standardized tests to measure psychological state, cognitive performance, and physiological condition to link functions to microbes; e) utilize human studies to measure psychological state, cognitive performance, physiological condition after stress (sleep deprivation), measurement of microbial metabolites, and treatments to test hypotheses (pre−/pro−/antibiotics; membrane vesicles, microbial metabolites); and f) support the development of novel tools for real-time analysis of microbes, the surfaces they adhere to, and microbial products in the GI tract to more closely map heterogeneity and identify links to function.

Dr. J. Philip Karl presented an overview of the collaborative gut microbiome research efforts and related capabilities at USARIEM [36] and NSRDEC [37] co-located at the NSSC, Natick MA. Collectively, this team has initiated a multi-disciplinary research program that aims to identify nutrition-based strategies for optimizing resiliency in the gut microbiome to military-relevant stressors. Dr. Karl began by providing a high-level overview of the USARIEM-NSRDEC team’s joint research program. The initial phase of the program was described as a nascent effort aiming to characterize the effects of military-relevant operational stressors on gut microbiota composition, function and activity, and to elucidate the consequences on Warfighter health and performance. Relevant operational stressors were described as including dietary intake, under-nutrition, sleep deprivation, psychological stress, prolonged physical activity, dietary supplements and medications, and environmental extremes (e.g., heat, altitude). Performance was defined as primarily acute effects on physical and cognitive function. Dr. Karl then described the objective of the program’s second phase, which is to identify pre-, intra- or post-exposure nutritional interventions for mitigating adverse effects of operational stressors on gut microbiome composition and activity. The latter portion of the presentation highlighted how the unique capabilities of the USARIEM and NSRDEC partners will be integrated. The research mission of the Military Nutrition Division, USARIEM is to conduct research that provides a biomedical science basis for developing new rations, menus, policies and programs that enable Warfighter health-readiness and optimal performance. Dr. Karl highlighted several ongoing human subject trials in which dietary intake (e.g., military ration consumption) and environmental conditions (e.g., military combat operations training) [38] were being manipulated to elucidate effects on gut microbiota composition and activity, and gut barrier function. Dr. Karl briefly described the importance of the gut barrier in maintaining a selective boundary between the host and exogenous compounds [39, 40], and cited evidence suggesting that military-relevant stressors could compromise Warfighter health and performance by altering gut barrier permeability [41, 42]. Further, the gut microbiome was identified as a malleable target for building resiliency to such stressors, and dietary intake was identified as the tool that would be utilized by the USARIEM-NSRDEC team to manipulate the gut microbiome in future studies. Dr. Karl then transitioned to noting one of the WD, NSRDEC research missions, which is to develop physiologically-relevant in vitro fermentation capabilities to provide models for understanding nutrient-gut microbiome interactions. Dr. Karl highlighted recent work by that team in which the gut microbiomes of human donors were maintained in an in vitro batch fermentation system to study microbe-mediated metabolism of cranberry polyphenols [43]. Dr. Karl continued by describing how NSRDEC in vitro fermentation capabilities will expand into a continuous fermentation system capable of replicating the environment within the ascending, transverse and descending regions of the human colon [44]. Physiologic relevance of the model will be enhanced through collaboration with the Combat Feeding Directorate, NSRDEC, wherein cell culture capabilities provide a means of challenging human cell lines with fermentate generated during in vitro fermentation experiments. Coupling in vitro mammalian intestinal cell culture models with in vitro fermentation experiments will allow basic mechanistic studies of pathways relevant to inflammation, innate immunity, and gut permeability. This platform will be integrated into the USARIEM-NSRDEC research program as a cost-effective, high-throughput means for screening candidate nutrients and nutrient combinations for their ability to restore gut microbiome homeostasis following stressor-induced perturbations and for their potential to enhance resiliency of the gut microbiome to such stressors. Dr. Karl concluded by elaborating on the mission of the CFD, NSRDEC, which includes identifying and developing feeding solutions for all military service branches. This critical position will provide opportunities for transitioning the knowledge gleaned from independent and joint USARIEM-NSRDEC human and in vitro studies into nutritional interventions that will subsequently be evaluated in future human trials.

Dr. Nancy Kelley-Loughnane, of the 711 Human Performance Wing (HPW), AFRL, presented an overview of how the microbiome and synthetic biology fits into the Air Force’s strategic science and technology vision of a ‘layered information’ concept of personalizing Airman health and performance. In order to address the Air Force’s future needs as set forth in Air Force Future Operating Concept (September 2015) and Global Vigilance, Global Reach, Global Power for America, 711 HPW has developed cross cutting research primary mission areas in order to synergize its research and development and medical efforts. The four primary mission areas (PMAs) include Airman Machine Teaming, Education-Training, Force Protection, and Airman Health and Performance. Understanding the microbiome and manipulating it via synthetic biology approaches will play an important role in the PMAs, especially Airman Health and Performance, which is defined as “the convergence of science, medicine, and engineering to enhance, optimize, and sustain the physical, psychological, cognitive, and behavioral states across the Airman’s lifecycle to achieve airpower dominance.” The 711 HPW expertise in biological sciences and performance measurement places it in a unique position to revolutionize personalized performance optimization for the Airman.

Ongoing microbiome-related efforts within the 711 HPW were highlighted that explore how microbiomes are modified by environmental conditions and how they might be modified to augment human performance. The Office of Naval Research-funded study, led by Dr. Victor Chan, is investigating how sleep-deprivation affects host intestinal function and gut microbiota composition and metabolism. This will allow development of a host-microbe metabolic interaction model for sleep deprivation, enabling in silico testing of potential strategies to modulate gut microbiota to counter the effects of sleep deprivation. Studies led by Dr. Camilla Mauzy at 711 HPW are investigating the effect of toxicants on the microbiome of the gut and the lung, and how this affects host epidermal permeability. These data will be used to determine if specific microbiome signatures can predict exposure and health. Finally, Dr. Michael Goodson, also from 711 HPW, highlighted an Air Force School of Aerospace Medicine-funded project that is investigating the role of the gut microbiome in deployment-associated diarrhea. The broad goal of this research is to identify components of the gut microbiome that are protective against diarrheal infection so that they can be used as a basis for prophylactic treatment in deployed personnel to reduce the incidence of diarrheal disease during and after deployment.

Overall, the 711 HPW team along with its service and academic partners are addressing the need to understand and the possibility of controlling the microbiome in order to improve Airman Health and Performance.

### Day two: Group breakout sessions

Day two of the information meeting featured working group breakout sessions to identify the following within the DoD: 1) current capabilities; 2) research areas of interest; 3) rationale for gut microbiome research and potential impact on the Warfighter; 4) fundamental and applied knowledge gaps; 5) resource gaps; 6) future research directions. Two parallel sessions were held in the morning that focused on programmatic issues, followed by two parallel afternoon sessions that focused on technical aspects of microbiome research. The programmatic sessions described what is needed to create an environment for success in DoD gut microbiome research. The technical sessions discussed the resources needed to execute these research programs.

## Programmatic perspectives for DoD gut microbiome research

Discussion on programmatic perspectives was structured around the knowledge that could be gleaned from microbiome research, the impact and implications of the knowledge to the Warfighter, and how that knowledge could be translated into strategies and products for improving Warfighter performance. Open discussions centered on how Warfighters are exposed to multiple unique stressors potentially impacting the microbiome, often in combination, and that gut microbiome research should largely focus on interactions between such stressors, the gut microbiome, and Warfighter health and performance. This focus should complement and build upon, rather than duplicate, the gut microbiome efforts being funded by the NIH and similar organizations, which largely emphasizes disease outcomes and chronic health. Avoiding duplication of efforts within the DoD was also discussed as necessary to maximize resources, knowledge generation and technology transition. To that end, it was suggested each service branch should consider establishing specific expertise within their research programs, making that expertise available to other service agencies, and communicating their research goals, directions and outcomes. This effort could include establishing a centralized infrastructure for some research activities, such as high-performance computing. However, it was recognized that some unique resources are not likely to be replicated within DoD laboratories. As such, partnering with academic institutions and industry to meet gut microbiome-related research goals will continue to be necessary.

Research towards “purposeful manipulation” of the gut microbiome will likely result in the development of novel strategies for improving Warfighter health and performance. The panel envisioned research aimed at engineering the gut microbiome by introduction of bacteria into the ecosystem (e.g., probiotics), targeting bacteria currently in the ecosystem through nutritional modulation, or via genetically engineered novel microorganisms. The goals identified for gut microbiome manipulation were myriad and included stimulating production of metabolites to enhance cognition and emotional state, modulating immune responsiveness to vaccines, promoting resistance to chemical attack, use of personalized medicine to inform intervention strategies, and reducing chronic disease risk. The microbiome was also seen as a predictive tool for performance and chronic health that could garner information for proactive decision-making to assist in determining the microbiome response for certain tasks or resistance to stressors. Proposed 10 year research goals included development of foods or identification of supplements targeting the gut microbiome that could be incorporated into military feeding scenarios. Additionally, introducing genetically engineered microbes into the human gut microbiome was considered a 20 year and beyond research objective.

Discussion also centered on the types of studies and outcomes needed to elucidate the role of the gut microbiome in Warfighter health, and to identify effective approaches for leveraging the gut microbiome to benefit the Warfighter. One current limitation is a lack of biological sample repositories for fecal, urine and blood samples from new recruits and Warfighters throughout their military careers. Extensive discussion centered on the utility of establishing policies for collecting and storing biological samples, recording Warfighter health information, being in compliance with guidelines for human use research, and creating protocols for retrieval of health information by various organizations. These facilities and repositories would facilitate prospective cohort studies, the lack of which was identified as a programmatic gap. Such studies would enable investigations into the long-term impact of military-relevant environments on the gut microbiome (e.g., pre- and post-deployment) and the relation to Warfighter health. It was suggested that programmatic initiatives could also focus on improving current methods of obtaining and recording health and other information from Warfighters. Current in-theatre information records were noted as incomplete, thereby creating gaps in Warfighter health timelines. Few widely accepted standards exist for conducting gut microbiome research. Therefore, establishing standard protocols for sample collection and analysis is a prerequisite to establishing biospecimen repositories and large databases. This need is corroborated by a recent study investigating the influence of sample collection and handling on detection of bacterial taxa via qPCR, which correlated multiple parameters to DNA degradation [45]. Another standardization aspect to consider is next-generation sequencing, typically through 16S rRNA sequencing. Although a recent study has demonstrated that multiple bioinformatics pipelines can identify similar bacterial composition within human fecal samples from 16S rRNA sequencing data [46], the influence of the multitude of variables within the 16S rRNA sequencing technique, including extraction techniques, on the data sets generated for bioinformatics analysis is unclear. As such, a programmatic focus on protocol standardization was suggested. Subsequent to this information meeting, the National Institutes of Standards and Technology hosted, from 9 to 10 August 2016, a Standards for Microbiome Measurements Workshop to discuss standardizing methods for gut microbiome research [47].

A separate, but related programmatic issue identified was the need to validate, define and standardize models and the outcomes used to measure the effects of interventions. In order to translate research, establishing appropriate animal and in vitro models to complement human studies is critical. Currently there is a lack of validated robust models within DoD laboratories, although several groups (e.g. rat models at AFRL, humanized-gut microbiota mouse models at USACEHR, and in vitro fermentation models at NSRDEC and AFRL) are actively developing promising models that can be leveraged by the DoD research community. Research outcomes would be significantly enhanced via use of standardized models. An additional barrier to translating gut microbiome research is the fact that challenges posed by inter-individual differences and environmental factors can impede detection of intra-individual variances over time in response to stress (e.g., sleep circadian disruptions), highlighting the importance of longitudinal studies for the duration of stressor exposure. In addressing these issues, it was agreed that systems-level approaches that integrate and correlate multiple biological sample matrices (e.g., blood, urine, fecal, tissue, saliva, etc.) would facilitate knowledge discovery and translation. The panel agreed that collaboration in using these models and incorporating standardized techniques and measurements will ultimately enhance DoD gut microbiome research.

The panel recommended establishing a DoD Tri-Service Microbiome Consortium (TSMC) to promote coordination and collaboration of gut microbiome research and to provide a forum for sharing ideas, research activities, resources and data. In order to improve collaboration and research progress, it was recognized that capabilities need to be identified across various organizations. Increasing awareness of existing capabilities within organizations and the development of novel capabilities was recognized as having a clear benefit to DoD gut microbiome research. Finally, it was recognized that microbiome research is resource intensive, requires significant funding, and that one of the primary challenges to existing research includes the current availability and stability of funding. Sharing of resources and leveraging programs such as MURIs, Small Business Technology Transfer (STTR)/Small Business Innovative Research (SBIR), Laboratory University Collaboration Initiative (LUCI), and Defense University Research Instrumentation Program (DURIP) will be necessary. Several participants noted that requirements stated in DoD capability documents are particularly influential in setting research priorities and funding, and that funding is often determined by threat; however, at present, there is no established direct threat related to the human microbiome, although microbiome-related interventions are possible solutions. A focus of the TSMC would be championing the need for and importance of gut microbiome research within the DoD, and identifying and securing funding where appropriate.

## Technical aspects of current and future DoD gut microbiome research

Discussion of technical issues centered on the mechanics for achieving near- and long-term research objectives in relation to existing technical resources, gaps, barriers, and projected research efforts. Overall, there are sufficient technological resources for developing tangible gut microbiome targeted solutions in the near-term. However, this research, and related capabilities, should also advance to inform future research and develop capabilities that facilitate achievement of longer term research objectives.

Several established DoD research and development capabilities offering potential for near-term knowledge transition were discussed. These proficiencies included food product development, access to Warfighter populations in austere environments and controlled settings, an ability to collect, store and transport biospecimens, animal and in vitro models for studying mechanisms of host-microbiome interactions, 16S rRNA and metagenomic sequencing capabilities, and bioinformatics expertise. These capabilities collectively enabled potential manipulation of the microbiome using pre-, pro- and synbiotics, case-control studies for identifying signatures of health and dysbiosis within the gut microbiome, longitudinal studies of gut microbiome responses to military-relevant stressors for both acute (sleep, under-nutrition, fatigue, diet, etc.) and chronic (PTSD, anxiety, depression, prolonged sleep deprivation, etc.) scenarios, the persistence of these effects, and the ability to build resistance to infection. However, it was noted that fundamental knowledge gaps impede interpretation of results from these studies and results are constrained. To address these gaps, new and improved methodologies are needed. The most immediate identified need was an improved understanding of mechanisms of host-microbiome and microbe-microbe interactions, including characterization of the role of fungi and viruses within the gut microbiome. One approach involved model systems (e.g., designed bacterial communities), rather than targeting the whole host-microbiome system, to generate more targeted and interpretable data. In parallel, integrated multi-omic (e.g., metagenome, metatranscriptome, metabolome, proteome, lipidome) approaches to studying dynamic environment-host-microbiome interactions were also suggested. Refining and standardizing current protocols for fecal sample collection [48, 49] and analysis [50–52] should be extended to approaches that may be more amenable to the austere environments in which Warfighters operate. In addition, technologies are also needed for real-time and non-invasive spatiotemporal sampling throughout the gastrointestinal tract to provide more relevant samples for both human and animal studies. Several panelists expressed concern over the translatability of animal models, particularly murine models in gut microbiome research. However, although animal models may not always translate to human outcomes, especially regarding human performance and cognition, they are, nonetheless, integral to developing mechanistic insights.

Interpretation of human and animal studies and the design of subsequent studies would be facilitated and enhanced by next generation computational tools/platforms for complex consortia including predictive modeling utilizing multi-omic readouts from organisms exposed to different military-relevant stressors and interventions. Such models would enable prediction of testable strategies for building gut microbiome resiliency and deemed development of such models as likely feasible within 10–15 years. Within the same time frame, the panel posited that better correlation of in vitro and animal models to human studies will be established. The state of gut microbiome research in 20 years and beyond was briefly discussed and it was thought that a systems biology approach would predominate in this area [53]. Moreover, the interaction of the gut microbiome with other microbial communities on the human body (e.g., lung, oral, vaginal, skin) is likely to emerge as relevant to Warfighter health and performance. Further, designer bacteria that were engineered to produce metabolites of interest or were able to sense and respond to the environment in order to manipulate and modulate the microbiome would also be desirable.

Technological discussions considered relevant physiological outcomes for DoD nutrition and gut microbiome research and how to measure them. Relevant outcomes fell into three broad categories: 1) maintaining gut resiliency; 2) optimizing function to improve performance; 3) restoring function after dysbiosis. Measuring resistance to infection should be a top priority for future research as building gut microbiome resiliency to pathogens would directly reduce lost duty time and improve Warfighter readiness. Increased energy and reduced fatigue were short term objectives that may be potentially relevant to gut microbiome homeostasis. During such efforts, multiple biological sample matrices, including real-time tissue/intestinal lining sampling, if possible, will need to be collected and cohesively analyzed to identify mechanisms underlying host-microbe communication. Understanding differences within individual gut microbiomes of responders and non-responders to a stressor or intervention is also critical. Studies should be conducted and outcomes measured in settings closely mimicking mission and/or training environments with detailed cohort studies as a vehicle to both gain knowledge and facilitate translation of data for targeted solutions. Such studies would be aided by the development of novel methods for real-time data assessments.

## Conclusions and recommendations

The 1st DoD Gut Microbiome Informational Meeting on nutrition and gut microbiome research included presentations and discussion among DoD scientists working in the field. Attendees agreed that the microbiome is highly relevant to Warfighter health and performance, and continued research is critical to mission readiness. Several common themes emerged during the meeting: 1) a need for increased collaboration, coordination, and communication, possibly via a central portal 2) a need to establish longitudinal studies of incoming recruits prior to initial military training, as well as pre- and post-deployment 3) although bioinformatics resources exists, the area was identified as a bottleneck that could be remedied by increasing bioinformatics expertise within the DoD 4) gut microbiome research is a relatively new field and rapidly evolving; thus research results must be interpreted within the context of the limitations to the methods used to collect the data, and staying abreast of technological advances is critical 5) many capabilities and resources required for gut microbiome research currently exist within the DoD; 6) collaborative use of these capabilities and resources in addition to method standardization will facilitate the translation/interpretation of results from different studies across the DoD, and ultimately research impact. Also identified were the potential DoD-specific barriers to gut microbiome research, such as funding, security clearances when working with non-DoD partners, contracting requirements that may impede collaboration, and meeting attendance and cybersecurity restrictions. Since 2015, progress has been made in a number of these areas: 1) steps to improve communication, collaboration and coordination have occurred as evidenced by establishment of the DoD Tri-Service Microbiome Consortium (TSMC) and its inaugural workshop held in May 2017; 2) interagency efforts have been undertaken to coordinate and streamline handling of large data sets from bioinformatics analyses (e.g. DoD cloud); and 3) the NIST/NIH sponsored Standards for Microbiome Measurements Workshop held in August 2016 met to prioritize needs for developing standards for microbiome measurements that will enable federal, academic, and industry labs to reliably reproduce and advance research.

**Fig. 3:**
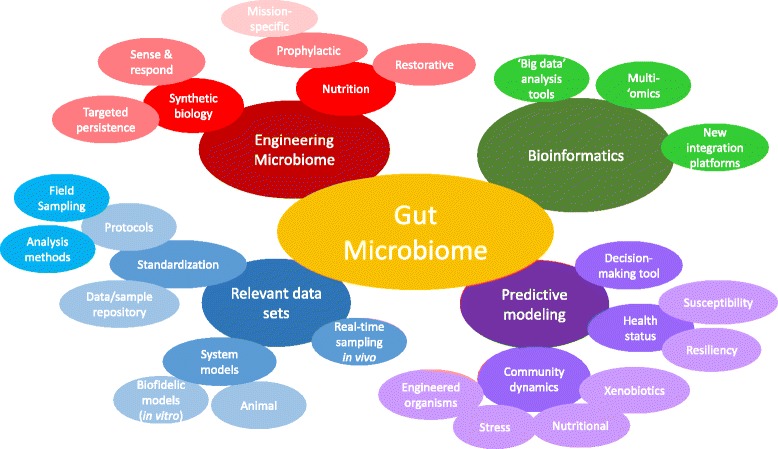
Several research thrust areas (large, dark ovals) emerged across the break-out sessions that can be further refined into sub-focus areas (medium ovals) and research concepts (smaller, outer ovals) to address the challenges in working toward common research areas of interest identified during the informational meeting

Over the course of the meeting, common research interests were evident, including understanding the influence of military-relevant stressors on interactions between the microbiome and Warfighter biology, manipulating the microbiome to influence Warfighter health, and using the microbiome as a biomarker of Warfighter health status. Current DoD research efforts appear complimentary rather than duplicative. Toward addressing common research interests, several key focus areas emerged across the technical and programmatic break-out sessions (Fig. 3).

Four research thrust areas – Engineering the Microbiome, Bioinformatics, Relevant Data Sets, and Predictive Modeling – were identified as research domains that are essential for both near- and long-term Warfighter solutions. Each of the areas can be broken down into multiple layers of sub-focus areas that entail specific approaches to addressing key challenges within the main research thrust. For example, two key approaches were identified as a means to engineer the microbiome - synthetic biology and nutrition. The synthetic biology approach can further be distilled into two main research concepts – designer organisms for targeted persistence and/or for sense/respond functions. Both concepts fall within the research interest areas of manipulating the microbiome and using the microbiome as a biomarker for health status. The additional research thrust areas similarly break down into sub-focus areas and link to the common areas of research interest. The identification of key research thrust areas and concepts to address technical/programmatic aspects of those areas was a key outcome of the meeting and serves as a starting point for establishing DoD gut microbiome research directions.

**Fig. 4:**
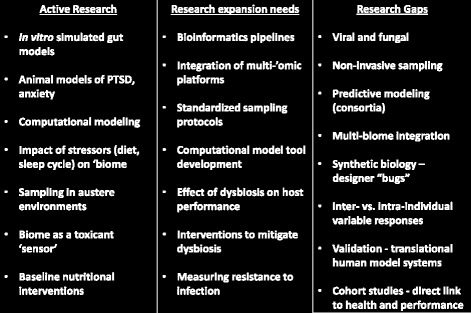
Current state of the science derived from the informational meeting. The current state includes active areas of research across the attendees, areas that are ongoing but need expansion through additional resources, capability development and novel techniques, and also research gaps that emerged as critical to providing translatable solutions to the Warfighter for improving health and performance. While efforts have been made to address these research areas and gaps, they remain relevant today

An objective of the meeting was to gain an understanding of the current state-of-the art at that time in DoD gut microbiome research and identify research gaps, as they pertain to the common research interest across the Services. Toward this goal, information was compiled to summarize the active DoD gut microbiome research, which areas of active research need expansion, and where the current gaps exist within the collective Services, as can be seen in Fig. 4. While work in these areas and gaps have been ongoing, they remain relevant today.

Active research across the Services center on developing tools and capabilities to gain baseline knowledge on the role of the gut microbiome in Warfighter health and performance. Current active areas in need of expansion represent program aspects that are at sufficient levels now to derive baseline knowledge. However, attendees recognized the need for enhancement in tools, capabilities, and, in some areas, research focus in order to generate more in-depth knowledge. Lastly, research gaps were identified as areas determined as essential but in which very limited or where an absence of current investigation was evident. The information gathered is not all inclusive, as the gut microbiome area is dynamic and new gaps or new research efforts can readily emerge; however the information can serve as a starting point for a collaborative, joint roadmap as DoD gut microbiome research programs are developed.

**Fig. 5:**
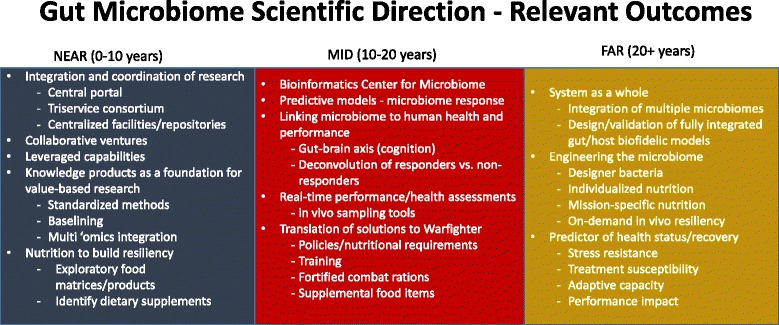
A compilation of the information across the 2-day meeting identified possible relevant outcomes of DoD gut microbiome research for the near-, mid-, and far-term. Identification of the possible outcomes assists in developing scientific direction for the area, however, additional assessment is still necessary

Although consensus could not be generated on a number of technical aspects, it was agreed that translating solutions to the Warfighter is the ultimate outcome of DoD gut microbiome research. Relevant research outcomes were identified as being extremely important to articulate to stakeholders and decision makers. Toward this end, the 2-day meeting information was compiled to generate near- (0–10 year), mid- (10–20 year) and far-term (20 year +) possible relevant outcomes for gut microbiome research **(**Fig. 5**)**.

Near-term outcomes center on two primary aspects: 1) avoiding duplicity in both capability and tool development as well as research focus, and, when necessary, avenues to effectively communicate and leverage complementary studies across all Services and 2) generation of novel platforms and standardization of methods and analyses that will facilitate the translation/interpretation of results from different studies across the DoD. Mid-term outcomes focus on those research areas that have been identified as necessary to expand to advance understanding of the complex interactions within the gut microbiome, including computational tool advancements and real-time data/sample acquisition. It is also feasible to extend focus along specific axes in which the gut plays a pivotal role. Ultimately, in the mid-term, scientific direction should center on translating solutions to the Warfighter to realize the benefits of using the gut microbiome to modulate health and performance. Far-term outcomes are more difficult to articulate due to the nascent nature of gut microbiome research in the DoD. However, relevant outcomes may center on looking at a more holistic level and focus primarily on engineering the microbiome for tailored resiliency on an individualized basis that can extend to mission-specific nutrition, utilizing models to predict health status and potential impact on performance. To realize the relevant outcomes, DoD gut microbiome research must be conducted through a coordinated, collaborative research environment that not only reaches throughout the Services but also includes academic, industrial and other relevant partners. Since 2015, several near-term outcomes have been advanced: 1) the chartering of the TSMC and its inaugural workshop sought to improve integration and coordination of research efforts, and 2) leveraging opportunities and collaborative efforts will be advanced by establishment of the Applied Research for the Advancement of S&T Priorities (ARAP) Program in Synthetic Biology for Military Environments (SBME) that will result in by building DoD capabilities in synthetic biology.

Although it was hoped that explicit technical recommendations would be realized during the meeting, breakout panels did not come to a consensus on all technical needs and gaps or approaches to address those topics. This is not uncommon to the field, and indicates a need for continued communication within the DoD and the broader scientific community. The immediate and foremost outcome of this meeting was the identification of DoD organizations active in gut microbiome research and complementary interests and capabilities. Furthermore, inter-institute and inter-agency coordination and communication was improved and/or established, which will allow collaboration within the DoD to leverage capabilities and enhance productivity, which collectively will facilitate working toward the desired relevant outcomes within gut microbiome research. The December 2016 establishment of the TSMC clearly serves as a powerful example of the motivation and momentum of the DoD microbiome community to coordinate and leverage the microbiome as a tool for improving the health and performance of the Warfighter. The outcome of the TSMC workshop in May 2017 determined that methods and analysis standardization, big data sharing and synthetic biology are priority areas that apply to all microbiome research. Similar meetings were deemed necessary to be conducted on a regular basis to continue communication, further identify collaborative opportunities, and continue to refine/update the scientific direction for DoD gut microbiome research. There was overwhelming support for holding future gut microbiome informational meetings, and to integrate academic and industry partners within these discussions.

## Abbreviations

AFRL: US Air Force Research Laboratory
ARL: US Army Research Laboratory
ARO: US Army Research Office
CFD: Combat Feeding Directorate
CMSCFAT: Continuous Multifaceted Soldier Characterization for Adaptive Technologies
DoD: Department of Defense
DURIP: Defense University Research Instrumentation Program
FFRDC: Federally Funded Research and Development Center
GM: Gut Microbiota
HPW: Human Performance Wing
ISB: Integrative Systems Biology Program
LUCI: Laboratory University Collaboration Initiative
MDRO: Multi-Drug Resistant Organisms
MMT: Microbiome Modulation of Toxicity
MURI: Multidisciplinary University Research Initiative
NIH: National Institutes of Health
NMIG: Natick Microbiome Interest Group
NMRC: Naval Medical Research Center
NSRDEC: US Army Natick Soldier Research Engineering and Development Center
NSSC: Natick Soldier Systems Center
OASD(R&D): Office of the Assistant Secretary of Defense for Research and Engineering
ONR: Office of Naval Research
PTSD: Post-Traumatic Stress Disorder
SBIR: Small Business Innovative Research
STTR: Small Business Technology Transfer
TMM: Toxicant Modulation of the Microbiome
TSMC: Tri-Service Microbiome Consortium
UARC: University Applied Research Center
USACEHR: US Army Center for Environmental Health Research
USAMRIID: US Army Medical Research Institute of Infectious Diseases
USARIEM: US Army Research Institute of Environmental Medicine
WD: Warfighter Directorate
